# Association of aortic arch and aortic valve calcifications with cardiovascular risk in patients on maintenance hemodialysis

**DOI:** 10.3389/fcvm.2022.1053265

**Published:** 2022-12-06

**Authors:** Min-Tser Liao, Chia-Ter Chao, Chung-Kuan Wu

**Affiliations:** ^1^Department of Pediatrics, Taoyuan Armed Forces General Hospital Hsinchu Branch, Hsinchu, Taiwan; ^2^Department of Pediatrics, Tri-Service General Hospital, National Defense Medical Center, Taipei City, Taiwan; ^3^Division of Nephrology, Department of Internal Medicine, National Taiwan University Hospital, Taipei City, Taiwan; ^4^Division of Nephrology, Department of Internal Medicine, National Taiwan University College of Medicine, Taipei City, Taiwan; ^5^Graduate Institute of Toxicology, National Taiwan University College of Medicine, Taipei City, Taiwan; ^6^Division of Nephrology, Department of Internal Medicine, Shin Kong Wu Ho-Su Memorial Hospital, Taipei City, Taiwan; ^7^School of Medicine, Fu Jen Catholic University, New Taipei city, Taiwan

**Keywords:** aortic arch calcification, aortic valve calcification, major adverse cardiovascular events, maintenance hemodialysis, aortic arch calcification score

## Abstract

**Introduction:**

This study aimed to investigate the association of aortic arch calcification (AoAC) and aortic valve calcification (AVC) with major adverse cardiovascular events (MACE) and cardiovascular and all-cause mortality in patients on maintenance hemodialysis (MHD).

**Methods:**

This study enrolled 297 adult patients with end-stage kidney disease who were on MHD. They were divided into those with an AoAC score <2 without AVC (*n* = 70, 23.6%), those with an AoAC score <2 with AVC (*n* = 96, 32.3%), and those with an AoAC score ≥2 regardless of AVC status (*n* = 131, 44.1%). We analyzed the risks of MACE, cardiovascular and overall mortality among the three groups using Cox proportional hazard analyses. Survival probabilities were estimated using the log-rank test *via* the Kaplan–Meier method.

**Results:**

Kaplan–Meier analysis revealed that the MACE-free rate and the survival rates of cardiovascular and overall mortality were significantly higher in adult chronic hemodialysis patients with AoAC score <2 without AVC, followed by those with AoAC score <2 with AVC, and then those with AoAC score ≥2 (log-rank test; all *p* < 0.01). The grade of AoAC is a significant risk factor for MACE, cardiovascular mortality, and overall mortality after adjusting for age and gender Relative to AoAC score <2 without AVC, adult chronic hemodialysis patients with AoAC score ≥2 remained an independently significantly risk factor of MACE (adjusted hazard ratio, 2.17; 95% confidence interval 1.11–4.20; *p* = 0.023) after adjusting for age, sex, and all significant variables in baseline characteristics.

**Conclusion:**

AoAC grade was positively correlated with a higher risk of MACE and cardiovascular and overall mortality. Furthermore, the presence of AVC modified the adverse cardiovascular risk associated with AoAC in patients on MHD.

## Introduction

Cardiovascular disease, including ischemic heart disease, arrhythmia, heart failure, stroke, and peripheral arterial disease, is the leading cause of death in patients undergoing maintenance hemodialysis (MHD) (1). Risk factors for cardiovascular disease in patients undergoing hemodialysis include traditional and non-traditional risk factors, such as advanced age, diabetes mellitus (DM), dyslipidemia, hypertension, oxidative stress, inflammation, fluid overload, and vascular calcification (2). Calcification of the vascular tree and heart valves, a common complication in patients on MHD, may result from these risk factors. Early identification of these risk factors is crucial to reducing the calcification risk (3).

Chest radiography is a simple and non-invasive examination to evaluate intima-media aortic arch calcification (AoAC). AoAC has been recognized as an important risk factor for adverse outcomes in patients on dialysis (4). A previous meta-analysis indicated that AoAC significantly increased the risk of all-cause mortality by 44% and cardiovascular mortality by 130% among patients on MHD (5).

Aortic valve calcification (AVC) varies between 44 and 55% in patients on MHD (6). AVC plays a crucial role in cardiac structural changes and the progression of cardiovascular disease. Moreover, cardiac valve calcification occurs 10–20 years earlier in patients with chronic kidney disease (CKD) than in the general population, and its progression may be 10 times faster in patients with end-stage kidney disease (1). Relative to the pathogenesis of AoAC, the pathogenesis of cardiac valve calcification, including AVC, is less well known, even though it shares risk factors and pathogenic features with vascular calcification. Although AoAC and AVC are positively correlated in patients on MHD, very few studies have evaluated the ability of combined AVC and AoAC to predict clinical outcomes in patients on MHD. Therefore, we aimed to evaluate the relationship of AVC and AoAC and major adverse cardiovascular events (MACE) in patients on MHD. In addition, the predictive efficacy of AoAC and AVC for cardiovascular and overall mortality was analyzed.

## Materials and methods

### Study population

We conducted a retrospective study to assess the association of AoAC (viewed on chest radiography) and AVC (viewed on echocardiography) with MACE and cardiovascular and overall mortality in patients on MHD. Patients were enrolled in the hemodialysis center of Shin Kong Wu Ho-Su Memorial Hospital between 1 October and 31 December 2018. The inclusion criteria were as follows: (1) age ≥18 years and (2) hemodialysis for more than three months. Patients on MHD in the hospital who did not undergo chest radiography or cardiac echography during the period were excluded. The study was approved by the Institutional Review Board of the Shin Kong Wu Ho-Su Memorial Hospital (No. 20220713R). The requirement for informed consent was waived because of the retrospective nature of this study. The study protocol adhered to the Declaration of Helsinki.

### Evaluation of aortic arch calcification using chest radiography

Aortic arch calcification was assessed using the specific scale proposed by Chao et al. (6). The aortic arch visualized on the chest radiograph was divided into four sections by circumference, and the number of calcified sections was counted to yield the AoAC score. The severity of AoAC was quantitatively assessed based on the AoAC staging system in which stages 0–3 represent a scale of vascular calcification severity: grade 0, no visible calcification; grade 1, small spots of calcification or a single thin area of calcification of the aortic knob; grade 2, one or more areas of thick calcification; and grade 3, circular calcification of the aortic knob.

### Collection of demographic, medical, and laboratory data

After participant identification, we collected their clinical features, including the patient’s age, sex, body weight, vintage, presence of type 2 DM, hypertension, hyperlipidemia, coronary artery disease, peripheral artery disease, heart failure, chronic obstructive pulmonary disease, and malignancies. Laboratory parameters included serum albumin, aspartate aminotransferase, alkaline phosphatase, total cholesterol, triglyceride, fasting blood glucose, hemoglobin, platelet count, serum ferritin, transferrin saturation (TSAT), aluminum level, uric acid, sodium, potassium, ionized calcium, phosphorus, intact parathyroid hormone (PTH), and HD adequacy (Kt/V urea). Medication regimens included angiotensin-converting enzyme inhibitors/angiotensin receptor blockers, beta-blockers, calcium antagonists, diuretics, statins, oral antidiabetic medications, insulin and analogs, antiplatelets, and anticoagulants.

### Echocardiography

An experienced cardiovascular physician without prior knowledge of any patient information performed two-dimensional echocardiography. All echocardiographic data were acquired according to the guidelines of the American Society of Echocardiography and recorded by an experienced cardiologist blinded to the clinical details. AVC was defined as the presence of bright echoes of more than 1 mm on one or more cusps of the aortic valve.

### Outcome definitions

The primary endpoint was MACE, and the secondary endpoints were cardiovascular and overall mortality. MACE were defined as the occurrence of any of the following events: myocardial infarction, coronary revascularization, stroke, hospitalization for heart failure, or death from cardiovascular causes.

### Statistical analysis

Continuous variables are expressed as mean ± SD and categorical variables as numbers with percentages. Differences between groups were analyzed *via* analysis of variance for continuous variables and the χ^2^ test for categorical variables. Variables relevant to survival were identified using the univariate Cox proportional hazard method. Age, sex, and all significant variables in Table 1 were selected for further analysis using multivariate Cox proportional hazards models. The Kaplan–Meier method was used to estimate survival probabilities using the log-rank test. The impact of AoAC and AVC on patient’s clinical outcomes was also examined using the Kaplan–Meier analysis. Statistical analyses were conducted using IBM SPSS Statistics for Windows version 19 (IBM Inc.). In all analyses, statistical significance was set at *p* < 0.05.

**TABLE 1:** Baseline characteristics of patients on MHD based on the severity of AoAC and the presence of AVC.

	AoAC <2 without AVC	AoAC <2 with AVC	AoAC ≥2	*p*-Value
Number of participants	70	96	131	
Age (years)	66.4 ± 11.7	59.9 ± 12.5	73.3 ± 13.3	<0.001
Sex, female (%)	34 (48.6)	37 (38.5)	70 (53.4)	0.083
Weight (kg)	61.9 ± 14.0	61.2 ± 13.5	58.1 ± 13.1	0.097
Vintage (years)	7.4 ± 7.0	8.8 ± 7.8	8.1 ± 7.8	0.480
**Comorbidities (%)**				
Type 2 DM	32 (45.7)	46 (47.9)	58 (44.3)	0.862
Hypertension	57 (81.4)	75 (78.1)	109 (83.2)	0.625
Hyperlipidemia	40 (57.1)	53 (55.2)	70 (53.4)	0.878
Coronary artery disease	22 (31.4)	50 (52.1)	58 (44.3)	0.030
PAD	17 (24.3)	20 (20.8)	43 (32.8)	0.112
Heart failure	10 (14.3)	21 (21.9)	32 (24.4)	0.241
COPD	5 (7.1)	10 (10.4)	11 (8.4)	0.748
Malignancy	7 (10.0)	11 (11.5)	13 (9.9)	0.924
**Laboratory data**				
Albumin (gm/dl)	3.9 ± 0.3	4.0 ± 0.3	3.8 ± 0.4	0.001
A.S.T. (IU/L)	16.3 ± 5.4	16.8 ± 5.7	16.5 ± 6.5	0.939
Alkaline-P (IU/L)	71.9 ± 44.8	69.2 ± 28.9	80.6 ± 41.4	0.072
Cholesterol (mg/dl)	157.6 ± 38.3	154.7 ± 38.3	158.2 ± 40.0	0.796
Triglyceride (mg/dl)	161.7 ± 148.7	128.4 ± 87.2	147.3 ± 97.6	0.146
Fasting glucose (mg/dl)	109.9 ± 53.9	104.3 ± 35.4	121.6 ± 66.7	0.061
Hemoglobin (g/dl)	10.7 ± 1.5	10.5 ± 1.4	10.2 ± 1.2	0.024
Platelet (×1,000/ul)	199.2 ± 56.7	184.2 ± 57.2	196.3 ± 58.6	0.181
Ferritin (ng/ml)	554.5 ± 268.1	538.8 ± 333.8	550.6 ± 250.6	0.929
TSAT (%)	34.1 ± 15.0	33.4 ± 14.1	28.9 ± 10.4	0.007
Al (ng/ml)	7.2 ± 4.5	6.5 ± 3.7	7.2 ± 4.2	0.395
Uric acid (mg/dl)	6.3 ± 1.7	6.2 ± 1.5	6.2 ± 1.7	0.883
Na (meq/L)	137.9 ± 2.9	138.1 ± 3.1	137.5 ± 3.2	0.444
K (meq/L)	4.8 ± 0.7	4.7 ± 0.6	4.6 ± 0.6	0.354
iCa (mg/dl)	4.5 ± 0.5	4.6 ± 0.5	4.6 ± 0.5	0.208
P (mg/dl)	5.3 ± 1.4	5.1 ± 1.2	5.0 ± 1.2	0.309
Kt/V (Gotch)	1.4 ± 0.2	1.4 ± 0.2	1.4 ± 0.2	0.472
PTH (pg/ml)	238.1 ± 196.2	250.6 ± 220.6	322.6 ± 323.9	0.048
**Medication (%)**				
**Anti-hypertensive drugs**				
ACEI/ARB	43 (61.4)	53 (55.2)	71 (54.2)	0.598
Beta-blockers	36 (51.4)	54 (56.2)	70 (53.4)	0.820
Calcium antagonists	40 (57.1)	59 (61.5)	75 (57.3)	0.786
Calcium-based phosphate binders	41 (58.6)	62 (64.6)	81 (61.8)	0.733
Non-calcium based phosphate binders	19 (27.1)	17 (17.7)	24 (18.3)	0.253
Calcitriol	26 (37.1)	44 (45.8)	54 (41.2)	0.526
Diuretics	15 (21.4)	24 (25.0)	29 (22.1)	0.832
Statins	31 (44.3)	30 (31.2)	47 (35.9)	0.224
OAD	16 (22.9)	34 (35.4)	46 (35.1)	0.153
Insulin and analogs	13 (18.6)	19 (19.8)	71 (54.2)	0.598
Antiplatelets	25 (35.7)	46 (47.9)	76 (58.0)	0.010
Anticoagulants	3 (4.3)	6 (6.2)	7 (5.3)	0.858

Data are expressed as *n* (%) for categorical variables and mean ± SD for continuous variables. MHD, maintenance hemodialysis; AoAC, aortic arch calcification; AVC, aortic valve calcification; DM, diabetes mellitus; PAD, peripheral arterial disease; COPD, chronic obstructive pulmonary disease; A.S.T., aspartate aminotransferase; TSAT, transferrin saturation; Al, aluminum; PTH, parathyroid hormone; ACEI/ARB, angiotensin-converting enzyme inhibitor/angiotensin II receptor blocker; OAD, oral antidiabetic medications.

## Results

**FIGURE 1:**
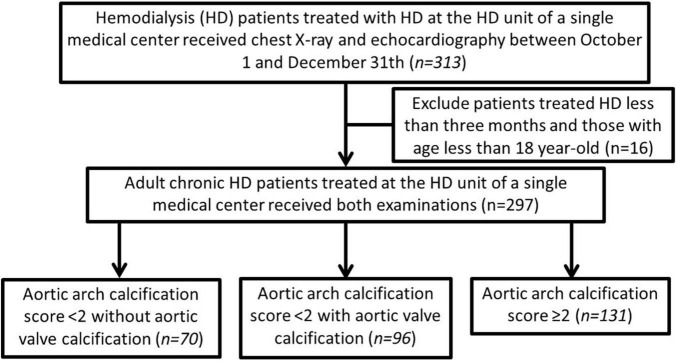
The participant selection process in this study.

A total of 313 patients with end-stage kidney disease receiving hemodialysis were screened, and 16 (5.1%) were excluded based on the exclusion criteria (Figure 1). A total of 297 adult patients on MHD were enrolled (156 men and 141 women), and 131 patients (44.1%) had AVC. Among the enrollees, 103 patients (34.7%) were scored as grade 0, 63 (21.2%) as grade 1, 79 (26.6%) as grade 2, and 52 (17.5%) as grade 3 of AoAC. The patients were stratified into three groups as follows: those with AoAC score <2 without AVC (*n* = 70, 23.6%), those with AoAC score <2 with AVC (*n* = 96, 32.3%), and those with AoAC score ≥2 (*n* = 131, 44.1%).

### Comparison of clinical characteristics and echocardiographic parameters among the study groups

A comparison of clinical characteristics among the study groups is shown in Table 1. Patients on MHD with an AoAC score ≥2 were significantly older (*p* < 0.001) and had a higher prevalence of coronary artery disease than those with an AoAC score <2, irrespective of the AVC status (*p* = 0.03). Regarding laboratory profiles, participants with an AoAC score ≥2 had the lowest albumin (*p* = 0.001), hemoglobin (*p* = 0.024), and TSAT (*p* = 0.007) levels but the highest intact PTH levels (*p* = 0.048) compared to the other two groups. Participants with an AoAC score ≥2 were more likely to use antiplatelet medications (*p* = 0.010) than those in the other two groups. We found that neither the phosphate binders nor calcitriol had significant associations with AoAC severity and presence/absent of AVC (*p* > 0.05).

We further examined the echocardiographic features in the 3 groups of participants. There was no significant difference in left ventricle (LV) mass, left ventricle end-systolic diameter (LVESD), and ejection fraction (EF) between the three groups (Table 2).

**TABLE 2:** Echocardiographic findings of MHD patients according to AoAC severity with and without AV calcification.

	AoAC <2 without AVC (*n* = 70)	AoAC <2 with AVC (*n* = 96)	AoAC ≥2 (*n* = 131)	*p*
Aortic root (mm)^§^	31.53 ± 4.23	31.98 ± 4.51	31.93 ± 4.75	0.643
IVS (mm)^§^	12.00 ± 2.55	11.66 ± 2.62	12.58 ± 4.96	0.322
LA diameter (mm)^§^	40.80 ± 7.79	43.29 ± 8.41	42.71 ± 7.70	0.242
LVEDD (mm)^§^	49.24 ± 7.91	50.21 ± 7.82	49.98 ± 7.49	0.410
LVESD (mm)^§^	31.67 ± 10.21	31.28 ± 8.79	31.46 ± 7.78	0.868
LVPW (mm)^§^	11.26 ± 2.41	10.68 ± 2.30	11.45 ± 3.31	0.131
LV mass (g)^§^	229.28 ± 82.13	223.96 ± 78.09	252.65 ± 197.87	0.684
LVMI^§^	138.45 ± 46.81	135.09 ± 42.78	159.59 ± 127.49	0.179
RWT (mm)^§^	0.47 ± 0.14	0.44 ± 0.16	0.47 ± 0.17	0.088
IVC diameter (mm)^§^	1.43 ± 0.41	1.55 ± 0.43	1.47 ± 0.43	0.198
EF (%)^§^	66.48 ± 13.93	68.19 ± 11.77	67.08 ± 11.27	0.683

Data are expressed as n (%) for categorical data and as mean ± SD for continuous data. AoAC, aortic arch calcification; CO, cardiac output; EF, ejection fraction; IVC, inferior vena cava; IVS, interventricular septum; LV, left ventricle; LVEDD, left ventricular end-diastolic diameter; LVESD, left ventricular end-systolic diameter; LVH, left ventricular hypertrophy; LVMI, left ventricular mass index; LVPW, left ventricular posterior wall; RWT, relative wall thickness; UCG, echocardiography.

^§^Kruskal–Wallis test.

### Risk of major adverse cardiovascular events and cardiovascular and overall mortality

**FIGURE 2:**
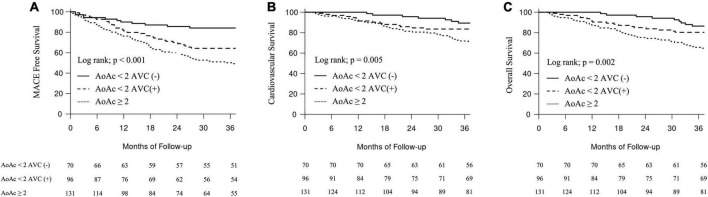
Major adverse cardiovascular events-free events and survival curves according to groups with different aortic arch calcification (AoAC) severity and aortic valve calcification (AVC). **(A)** MACE-free events. **(B)** Cardiovascular survival. **(C)** Overall survival.

Kaplan–Meier analyses were performed to examine the univariate association between the severity of AoAC and the presence of AVC and MACE and cardiovascular and overall mortality. After 3 years of follow-up, 91 (30.6%) patients on MHD developed MACE, among whom 14 (4.7%), 27 (9.1%), and 50 (16.8%) had an AoAC score <2 without AVC, <2 with AVC, and ≥2, respectively. Patients with an AoAC score ≥2 had a significantly higher incidence of MACE than those with an AoAC score <2, with or without AVC (log-rank test, *p* < 0.001) (Figure 2A).

Regarding secondary outcomes, 91 (30.6%) patients on MHD died owing to cardiovascular causes. Fourteen (4.7%), 27 (9.1%), and 50 (16.8%) patients died owing to cardiovascular causes with an AoAC score <2 without AVC, <2 with AVC, and ≥2, respectively. Patients with an AoAC score ≥2 had significantly worse cardiovascular and overall survival than those with an AoAC score <2 with or without AVC (log-rank test, *p* < 0.001; log-rank test, *p* = 0.002) (Figures 2B,C).

### Incremental values of aortic arch calcification and aortic valve calcification associated with the risk of major adverse cardiovascular events and cardiovascular and overall mortality

We conducted Cox proportional hazard regression analyses, with the development of MACE as the dependent variable, with and without incorporating variables with significant differences in the univariate analyses and variables with potential outcome influences. In the unadjusted analysis, the grade of AoAC [hazard ratio (HR) 1.43, 95% confidence interval (CI) 1.22–1.68] had a significantly increased risk of developing MACE. Patients with AVC (HR 1.95, 95% CI 1.26–3.03) had a significantly increased risk of developing MACE compared to those without AVC. Patients with an AoAC <2 with AVC (HR 2.42, 95% CI 1.26–4.64) also had a significantly increased risk of developing MACE compared to those with an AoAC score <2 without AVC and those with an AoAC ≥2 (HR 3.77, 95% CI 2.04–6.96) (Table 3).

**TABLE 3:** Cox proportional hazard analysis of outcome events in patients on MHD according to the severity of AoAC with and without AVC.

Events	Crude		Model 1		Model 2	
	HR (95% CI)	*p-*Value	HR (95% CI)	*p-*Value	HR (95% CI)	*p-*Value
**MACEs**						
Grade of AoAC	1.43 (1.22–1.68)	<0.001	1.34 (1.11–1.62)	0.003	1.19 (0.98–1.45)	0.080
Presence of AVC	1.95 (1.26–3.03)	0.003	1.52 (0.95–2.42)	0.079	1.38 (0.85–2.24)	0.196
AoAC <2 with AVC	2.42 (1.26–4.64)	0.008	1.98 (1.01–3.85)	0.045	1.81 (0.89–3.68)	0.102
AoAC ≥2	3.77 (2.04–6.96)	0.000	2.93 (1.51–5.69)	0.001	2.17 (1.11–4.20)	0.023
**CV mortality**						
Grade of AoAC	1.50 (1.21–1.87)	<0.001	1.37 (1.07–1.76)	0.013	1.21 (0.93–1.58)	0.153
Presence of AVC	1.49 (0.85–2.61)	0.162	1.04 (0.57–1.89)	0.896	1.06 (0.57–1.99)	0.856
AoAC <2 with AVC	1.62 (0.70–3.76)	0.259	1.18 (0.50–2.79)	0.706	1.49 (0.58–3.80)	0.405
AoAC ≥2	2.93 (1.39–6.16)	0.005	1.93 (0.86–4.36)	0.112	1.86 (0.76–4.52)	0.173
**Mortality**						
Grade of AoAC	1.45 (1.19–1.77)	<0.001	1.35 (1.07–1.70)	0.010	1.18 (0.94–1.50)	0.154
Presence of AVC	1.40 (0.87–2.27)	0.169	0.97 (0.59–1.62)	0.920	0.93 (0.55–1.56)	0.771
AoAC <2 with AVC	1.28 (0.63–2.60)	0.488	0.93 (0.46–1.90)	0.847	1.05 (0.49–2.21)	0.908
AoAC ≥2	2.53 (1.38–4.64)	0.003	1.64 (0.84–3.19)	0.149	1.40 (0.73–2.71)	0.313

AoAC, aortic arch calcification; AVC, aortic valve calcification; AoAC, Model 1, adjusted for age and sex; Model 2, adjusted for age, sex, coronary artery disease, albumin, transferrin saturation, PTH, and antiplatelets.

After adjusting for age and sex (Model 1; Table 3), the increase in AoAC grade (HR 1.34, 95% CI 1.11–1.62) was significantly associated with a higher risk of developing MACE. Patients with an AoAC <2 with AVC (HR 1.98, 95% CI 1.01–3.85) still had significantly higher risk of developing MACE compared to those with an AoAC score <2 without AVC and those with an AoAC score ≥2 (HR 2.93, 95% CI 1.51–5.69) (Model 1; Table 3). After adjusting for age, sex, and all significant variables in Table 1, patients with an AoAC score ≥2 (HR 2.17, 95% CI 1.11–4.20) remained significantly at risk of developing MACE compared to those with an AoAC score <2 without AVC.

Regarding secondary outcomes, Cox proportional hazard regression indicated that the increased AoAC grade was significantly associated with the risk of cardiovascular and overall mortality (HR 1.5, 95% CI 1.21–1.87; HR 1.45, 95% CI 1.19–1.77, respectively). Patients with an AoAC score ≥2 had significantly higher risk of cardiovascular and overall mortality (HR 2.93, 95% CI 1.39–6.16; HR 2.53, 95% CI 1.38–4.64) than those with an AoAC score <2 without AVC. After adjusting for age and sex (Model 1; Table 3), the increased AoAC grade still had a significantly higher risk of cardiovascular and overall mortality (HR 1.37, 95% CI 1.07–1.76; HR 1.35, 95% CI 1.07–1.70).

## Discussion

This study investigated the association of AoAC and AVC with MACE and cardiovascular and overall mortality in 297 patients on MHD. We found that patients with a higher AoAC score ≥2 exhibited the highest risk of MACE, followed by those with an AoAC score <2 and AVC. A similar trend was observed for secondary endpoints, including cardiovascular and overall mortality. Among those with an AoAC score <2, the presence of AVC developed the predictive ability for MACE in patients on MHD. Our study indicated that AoAC and AVC jointly modulated the risk of MACE in patients on MHD, and appropriate targeted strategies should be offered to mitigate these risk factors.

The Kidney Disease: Improving Global Outcomes (KDIGO) clinical practice guidelines recommend plain radiograph of the lumbar spine to assess cardiovascular calcification in patients on MHD (7). Accumulating evidence supports the additional value of the AoAC score assessed using chest radiography to predict outcomes. Komatsu et al. indicated that the presence and progression of AoAC are independently associated with mortality in patients on MHD (8). Lee et al. demonstrated that a high-grade AoAC score was associated with cardiovascular and overall mortality in 712 patients on MHD (9). The meta-analysis indicated that the presence of AoAC was associated with a greater risk of cardiovascular (HR 2.30; 95% CI 1.78–2.97) and all-cause (HR 1.44; 95% CI 1.19–1.75) mortality in patients on MHD (5). The pathogenesis of AoAC in patients on dialysis may involve both arteriosclerosis (arterial stiffening) and atherosclerosis (10). Hyperlipidemia, hypertension, metabolic syndrome, oxidative stress, and CKD are the major causes of endothelial injury, and disruption of endothelial-derived relaxing factors may indicate an early stage of atherosclerosis. Abnormal mineral metabolism, predominantly hyperphosphatemia, hypercalcemia, and deficiency of calcification inhibitor, facilitate atherosclerosis by transforming vascular smooth muscle into chondrocyte- or osteoblast-like cells with increased expression of core-binding factor A1 and osteopontin (11). In our study, patients with an AoAC score >2 also were significantly older; had lower serum albumin, low serum hemoglobin and TSAT levels; and elevated serum PTH levels because of malnutrition, inflammation, and atherosclerosis syndrome (Table 1) (12). Our study indicated that AoAC scores ≥2 were associated with increased MACE and cardiovascular and overall mortality (Figure 2). The AoAC grade was a strong risk factor for MACE and cardiovascular and overall mortality during the follow-up period (Model 1; Table 3).

Previous studies that included over 600 patients on dialysis reported a prevalence of AVC varying between 28 and 55% (1, 13). The annual incidence of AVC ranges from 1.5 to 8.0%, and the mean survival time after diagnosis is 23.0 ± 9.5 months (14). AVC and AoAC share common risk factors, including chronic inflammation, metabolic factors, and mineral and hormone-related factors in their pathogenesis (15, 16). Patients with vascular calcification also have a higher risk of developing AVC (17). Valvular calcification is commonly associated with hyperlipidemia and aging. Valvular calcification includes abundant subendothelial lipids and extracellular matrix, with a displacement of the elastic lamina in the early stage (18). Chronic elevation of inflammatory cytokines causes circulating osteoprogenitors to increase and induce endothelial-mesenchymal transition, resulting in cardiac valve calcification in patients on MHD (19). In our study, a lower AoAC score (<2) with AVC was associated with a risk of MACE in patients on MHD, and AVC likely played a modulatory role in this risk elevation (Table 3).

A recent meta-analysis also indicated that AVC was correlated with higher overall and cardiovascular mortality risk in patients on MHD (20, 21). A retrospective study by Li et al. showed that only AVC increased the risk of cardiovascular death in 183 patients on MHD (22). The cardiovascular risk-modulation effect of AVC can be interpreted in several aspects. In patients on MHD, cardiac output and heart rate increase in combination with anemia, creation of arteriovenous shunts, fluid overload, and electrolyte disturbance. Increased arterial pressure and blood flow over the stenotic aorta and vascular endothelial layer induce aortic wall shear stress and aortic stiffness and increase the risk of LV hypertrophy (23, 24). These mechanical factors may increase the oxygen demand and cardiac afterload. Further studies should investigate these influencing factors.

Other factors modulating the cardiovascular risk caused by AoAC have been previously reported. Ou et al. demonstrated that the radiographic-cardiothoracic ratio improved cardiovascular and overall mortality prediction in addition to AoAC in patients on MHD (25). Chen et al. indicated that radiographic abdominal aortic calcification and echocardiographic cardiac valve calcification were independently associated with mortality in patients on MHD (26). The risk of cardiovascular calcification is increased in patients on MHD owing to underlying disordered mineral and bone metabolism, chronic inflammation, and oxidative stress. The presence of AVC may intuitively signify a more aberrant response to AoAC and further enhance future cardiovascular risk.

Thus, our findings may be clinically important. Although the AoAC was not severe, we should aim to reduce the severity of AVC with lifestyle modification, proper blood pressure control using renin–angiotensin–aldosterone system blockers and statins, maintenance of normal vitamin D status, and proper supplementation with vitamin K (1). These strategies may further slow the progression of AVC and the residual risk of cardiovascular events in patients on MHD.

There were several limitations of this study. First, the evaluation of AoAC is semi-quantitative, which might underestimate accurate calcium deposition in the aortic wall. Second, the population size was small. Third, our study lacked the hemodynamic parameters on AoAC and AVC and the biomarkers for calcification. In the future, a larger multicenter prospective study is needed to divide the participants into subgroups of different features for result exploration.

## Conclusion

In conclusion, a high AoAC score (≥2) was a strong risk factor for MACE and cardiovascular and overall mortality. Among patients with lower AoAC on MHD, the presence of AVC further aggravated cardiovascular risk. Regular follow-up chest radiography and echocardiography could be simple and useful methods to stratify mortality risk in patients on MHD. Proper management of AoAC and AVC in this population should be emphasized to reduce future risk of adverse cardiovascular events.

## Data availability statement

The raw data supporting the conclusions of this article will be made available by the authors, without undue reservation.

## Author contributions

M-TL and C-KW: conception and design and writing the manuscript. M-TL, C-TC, and C-KW: analysis and interpretation, critical revision of the manuscript, and final approval of the article. C-KW: data collection, statistical analysis, and overall responsibility. C-TC: assistance in writing the manuscript. All authors contributed to the article and approved the submitted version.
